# Behavioral, Ventilatory and Thermoregulatory Responses to Hypercapnia and Hypoxia in the Wistar Audiogenic Rat (WAR) Strain

**DOI:** 10.1371/journal.pone.0154141

**Published:** 2016-05-05

**Authors:** Érica Maria Granjeiro, Glauber S. F. da Silva, Humberto Giusti, José Antonio Oliveira, Mogens Lesner Glass, Norberto Garcia-Cairasco

**Affiliations:** Physiology Department, Ribeirão Preto School of Medicine, University of São Paulo, 14049–900, Ribeirão Preto, SP, Brazil; University of Modena and Reggio Emilia, ITALY

## Abstract

**Introduction:**

We investigated the behavioral, respiratory, and thermoregulatory responses elicited by acute exposure to both hypercapnic and hypoxic environments in Wistar audiogenic rats (WARs). The WAR strain represents a genetic animal model of epilepsy.

**Methods:**

Behavioral analyses were performed using neuroethological methods, and flowcharts were constructed to illustrate behavioral findings. The body plethysmography method was used to obtain pulmonary ventilation (VE) measurements, and body temperature (Tb) measurements were taken via temperature sensors implanted in the abdominal cavities of the animals.

**Results:**

No significant difference was observed between the WAR and Wistar control group with respect to the thermoregulatory response elicited by exposure to both acute hypercapnia and acute hypoxia (p>0.05). However, we found that the VE of WARs was attenuated relative to that of Wistar control animals during exposure to both hypercapnic (WAR: 133 ± 11% vs. Wistar: 243 ± 23%, p<0.01) and hypoxic conditions (WAR: 138 ± 8% vs. Wistar: 177 ± 8%; p<0.01). In addition, we noted that this ventilatory attenuation was followed by alterations in the behavioral responses of these animals.

**Conclusions:**

Our results indicate that WARs, a genetic model of epilepsy, have important alterations in their ability to compensate for changes in levels of various arterial blood gasses. WARs present an attenuated ventilatory response to an increased PaCO_2_ or decreased PaO_2,_ coupled to behavioral changes, which make them a suitable model to further study respiratory risks associated to epilepsy.

## Introduction

Epilepsy, which affects 1–3% of the global population, is traditionally viewed as a neuropathological condition characterized by recurrent epileptic seizures that are associated with abnormal, excessive and/or hypersynchronous neuronal activity [1]. A more contemporary view on the epilepsies has to consider the increase risk to present seizures and their complexity, based upon networks, more than a localized circuit approach [2, 3], which is reflected, for example, on the concept of systems epilepsy [4].

Abnormal cardiac and respiratory functioning may occur during seizures; some of the malfunctions that may occur include cardiac arrhythmias and asystole along with severe breathing disturbances, including hypoventilation due to central apnea that ultimately leads to hypoxemia and hypercapnia [5,6]. Furthermore, a considerable body of evidence suggests that various chemoreceptor reflexes are dysfunctional in animal models [7] and in patients with epilepsy [8].

Although it is quite important to investigate the generation, processing, and modulation of the breathing and behavioral responses that are elicited by hypercapnia or hypoxia in seizing patients and experimental animals with epilepsy, or during interictal periods (temporal epochs with no seizure activity), we strongly need also to demonstrate how much patients and animals, are genetically-prone to seizures, with concomitant cardio-respiratory alterations.

The Wistar Audiogenic Rat (WAR) is a genetically-developed strain [9], which was selected by its response, in acute protocols with tonic-conic seizures and in chronic protocols (audiogenic kindling) with temporal lobe-like seizures [10,11,12]. We need, therefore, to emphasize that elucidating possible differences in the basal respiratory system and responses to central and peripheral chemoreflex activation in an epilepsy animal model such as the WAR strain, may not only be valuable for determining the genetic basis of variation in physiological behaviors, but also directly provide insight into the fundamental pathophysiological mechanisms involved in breathing control of patients with epilepsy.

Therefore, the objective of the present study was to evaluate the ventilatory, thermoregulatory and behavioral responses elicited to peripheral and central chemoreceptors activation in the WAR strain [9], without any seizure experience (naïve animals). Our hypothesis was that in genetically selected naïve WARs there would be behavioral, thermoregulatory and ventilator alterations in response to hypercapnia (7% CO_2_) and hypoxia (7% O_2_). Although our purpose is not to track any causal relationship, if present, these alterations, associated to WARs susceptibility to epilepsy, would be further studied as potential risk factors for example, Sudden Unexpected Death in Patients with Epilepsy (SUDEP) [5,6].

## Material and Methods

### Ethics Statement

All of the experimental protocols were reviewed and approved by the Animal Care and Use Committee (CETEA) at the Ribeirão Preto School of Medicine at the University of São Paulo (Protocol number 005/2011) prior to the start of the study.

### Animals

WAR animals were derived from a Wistar strain of albino rats and were bred selectively on the basis of their sensitivity to audiogenic seizures [9] at the Physiology Department of the Ribeirão Preto School of Medicine at the University of São Paulo. Male Wistar and WAR rats were used in the present study. Age-matched (30- to 40-day-old) WAR animals and control Wistar rats were individually housed in a controlled environment with a light/dark cycle of 12/12 h (lights were turned on at 6 a.m. and were turned off at 6 p.m.). Animals were given free access to standard rat food and water for the duration of the experiment.

### Surgery

All surgical procedures were performed under anesthesia following an intraperitoneal (IP) injection of 2,2,2-tribromoethanol (250 mg kg^−1^) and a preventive intramuscular injection of antibiotics (160,000 U kg^−1^ benzylpenicillin, 33.3 mg kg^−1^ streptomycin, and 33.3 mg kg^−1^ dihydrostreptomycin). A temperature sensor (datalogger, SubCue, Calgary, AB, Canada) was used to obtain body temperature (Tb) measurements and was implanted in the abdominal cavity via a midline laparotomy three or four days before the initiation of the experimental protocol. The datalogger in each animal was programmed to acquire temperature data every 5 min.

### Determination of pulmonary ventilation

Measurements of pulmonary ventilation (VE) were performed using the whole body plethysmography method [13]. VE was expressed either as absolute values (mL^.^ min^-1^) or relative to the body weight (mL^.^ min^-1.^ kg^-1^). Freely moving rats were kept in a 3.9 L chamber that was ventilated with either normoxic air (21% O2, N2 balance), with a humidified hypercapnic gas mixture (7% CO2, 21% O2, N2 balance) or with a hypoxic (7% O2, N2 balance) gas mixture. All of the gas mixtures, set at a flow rate range of 1200–1300 mL/min, were generated via a gas-mixing flowmeter (GF-3/MP Cameron Instruments Co, Port Aransas, TX, USA). During the VE measurements, airflow through the chamber was interrupted and the chamber sealed for a short period of time (~1 min). The subsequent breathing-induced pressure oscillations were monitored using a differential pressure transducer (ML141 Spirometer, PowerLab, ADInstruments, Bella Vista, NSW, Australia). Tidal volume (VT) was calculated according to the appropriate formula [14].

### Behavioral Analysis

Neuroethological methods were used to detect the frequencies, and durations of several behaviors, and to determine strengths of the statistical associations (chi-square) between pairs of behaviors. To evaluate behavioral sequences (cluster analysis), we used the ETHOMATIC software program [15] to measure observed dyadic (paired) behavioral interactions. Behavioral sequences were quantified on the basis of video recordings from which two aspects of the presentation times (latency and duration) of the behaviors were analyzed, as were the frequencies of various behaviors (see specific codes in the Results section). The behavioral frequency of each behavior was defined as the mean of the number of instances of that behavior that were detected during a specific observation period. Behavioral duration was defined as the mean duration of a given behavior over a given observation period [15]. Chi-square tests were used as a statistical measure of the strength of a given behavioral sequence. Flowcharts that were indicative of specific behavioral sequences were constructed in accordance with the statistically identified interactions of pairs of behaviors (dyads) that occurred in a given sequence in each experimental condition (normoxia, hypercapnia or hypoxia). Groups of behaviors among which there were significant associations constituted behavioral clusters and specific colors or circles around them were used only for representational purposes and did not impact the statistical analyses [10]. Behavioral items widely used in the Results section will be listed in the corresponding Dictionary. Details of the calibration and construction of the aforementioned flowcharts are illustrated in the corresponding Figure legend.

The behavioral analysis of the control phase (normoxia) of our experiment was performed using data that were recorded during the five minutes that immediately preceded the introduction of a hypercapnic or hypoxic atmosphere. The behavioral responses during hypercapnia or hypoxia were quantified in two different periods. The first of these occurred at the beginning of exposure to hypercapnia or hypoxia exposure; this window included minutes 0–5 of the experimental epoch. The second recording period occurred at the end of the period during which the animal was exposed to either hypercapnia or hypoxia; this temporal window comprised minutes 25–30 for the hypercapnic condition and minutes 55–60 for the hypoxic condition.

### Experimental Protocols

Measurements of VE and Tb were made simultaneously. The experimental protocol that was performed in the present study was adapted from one presented by Nattie and Li [16]. All experiments were carried out during the light period between 8 AM and 6 PM, and each rat was studied over a period of 4–5 h. Each rat was given at minimum of 4 days to recover from its surgery prior to any further data collection, and baseline values of VE, VT, respiratory frequency (fR), and Tb were measured for each rat while it breathed room air (normoxia), a hypercapnic gas mixture (7% CO2), and a hypoxic gas mixture (7% O2).

In all experimental protocols, each animal (either a normal Wistar rat or a WAR) was individually placed inside an open plethysmographic chamber and was allowed to adapt to the new environment for a minimum of 30–40 min before the experimental protocol was initiated. Then VE was recorded during a 1-2-min period of exposure to room air (a normoxic control period), after which the rats were exposed to a hypercapnic environment (7% CO_2_) for 30 min. Twenty min after the end of the period of exposure to hypercapnia, the chamber was closed again and another VE measurement was recorded to examine the recovery of the hypercapnic response. The plethysmographic chamber was then kept open to allow the animal to have a 120-min recovery period. The chamber was subsequently resealed and a second control measurement of the VE of the rat was taken during a 1-2-min period of exposure to room air (another normoxic control period). At the conclusion of this control period, animals were exposed to hypoxic environment (7% O_2_) for 60 min. Twenty min after the end of the period of exposure to hypoxia, the chamber was closed again and the breathing of the animal was recorded again to examine the recovery of the hypoxic response. VE values were also measured at several time points during the experimental conditions; during exposure to a hypercapnic environment, VE values were obtained at the 5-, 10-, 20- and 30-min time points. During exposure to hypoxia, VE values were measured at the 5-, 10-, 20-, 30-, 40-, 50- and 60-min time points.

### Statistical analysis

Data distribution was normal and was expressed as means ± SEMs. Measurements of VE, VT, fR e Tb before and after hypoxia or hypercapnia period were analyzed using two-way ANOVA with group (WAR hypoxia vs Wistar hypoxia or WAR hypercapnia vs Wistar hypercapnia) as the independent factor and time as the repeated measurement factor. When interaction between factors was observed, groups were compared by Bonferroni’s post hoc test. Basal values of body weight, Tb and ventilatory parameters during normoxia conditions (Table 1) and VE peak values (Table 2) were evaluated by unpaired Student’s t test. The measurement of statistical associations between pairs of behaviors (dyads), needed for the flowchart construction was made by means of transition matrices. The behavioral interactions were illustrated with arrows built proportionally to the statistical values when X^2^ > 3.84; P < 0.05). In fact the flowchart calibration used log X^2^ as the connector between behaviors [10,15].

**Table 1:** Basal values of body weight, Tb and ventilatory parameters of Wistar and WAR groups during normoxia conditions (control period), before hypercapnia or hypoxia.

	Wistar (n = 9)	WAR (n = 6)
**Body weight (g)**	237 ± 2	119 ± 5*
**Tb (**°**C)**	36.7 ± 0.1	36.3 ± 0.2
**fR (breaths/min)**	108 ± 4	104 ± 8
**VT (mL)**	1.6 ± 0.1	1.5 ± 0.08
**VE (mL/min)**	175 ± 10	157 ± 12

All values presented as mean ± SEM. Tb, body temperature, f_R_, respiratory frequency; V_T_, tidal volume; V_E_, ventilation.

^(^*^)^ Significantly different from Wistar control group. Unpaired Student’s t tests.

**Table 2:** The peak changes in V_E_ after hypoxia or hypercapnia of Wistar (n = 9) and WAR (n = 6) expressed in absolute values (mL^.^ min^-1^), relative to the body weight (mL^.^ min^-1.^ kg^-1^).

	Hypoxia		Hypercapnia	
	Wistar	WAR	Wistar	WAR
ΔV_E_ mL^.^ min^-1^	141±14	54±13*	239±29	58±13*
ΔV_E_ mL^.^ min^.^ kg^-1^	592±61	368±34*	919±78	569±92*

All values presented as mean ± SEM.

^(^*^)^ Significantly different from Wistar control group. Unpaired Student’s t tests. V_E_, ventilation.

## Results

### Body-weight Analysis

In accordance with recent studies from our laboratory [17] we observed that the mean body weight of the animals in the WAR group (n = 6) was significantly lower (p<0.05) than that of the Wistar animals (n = 9) (Table 1). Baseline respiratory parameters are shown in Table 2, and the ventilatory responses that were elicited by exposure to either hypercapnia or hypoxia (see below) are shown as percentages of the control (normoxia) responses to normalize the data between the two groups.

### Pulmonary ventilation and Tb Analysis

#### Normoxia

There were no significant differences in baseline parameters between control Wistar rats (n = 9) and WARs (n = 6) during the control period (before hypercapnia or hypoxia, p>0.05, Table 2).

#### Ventilatory responses to hypercapnia

**Fig 1:**
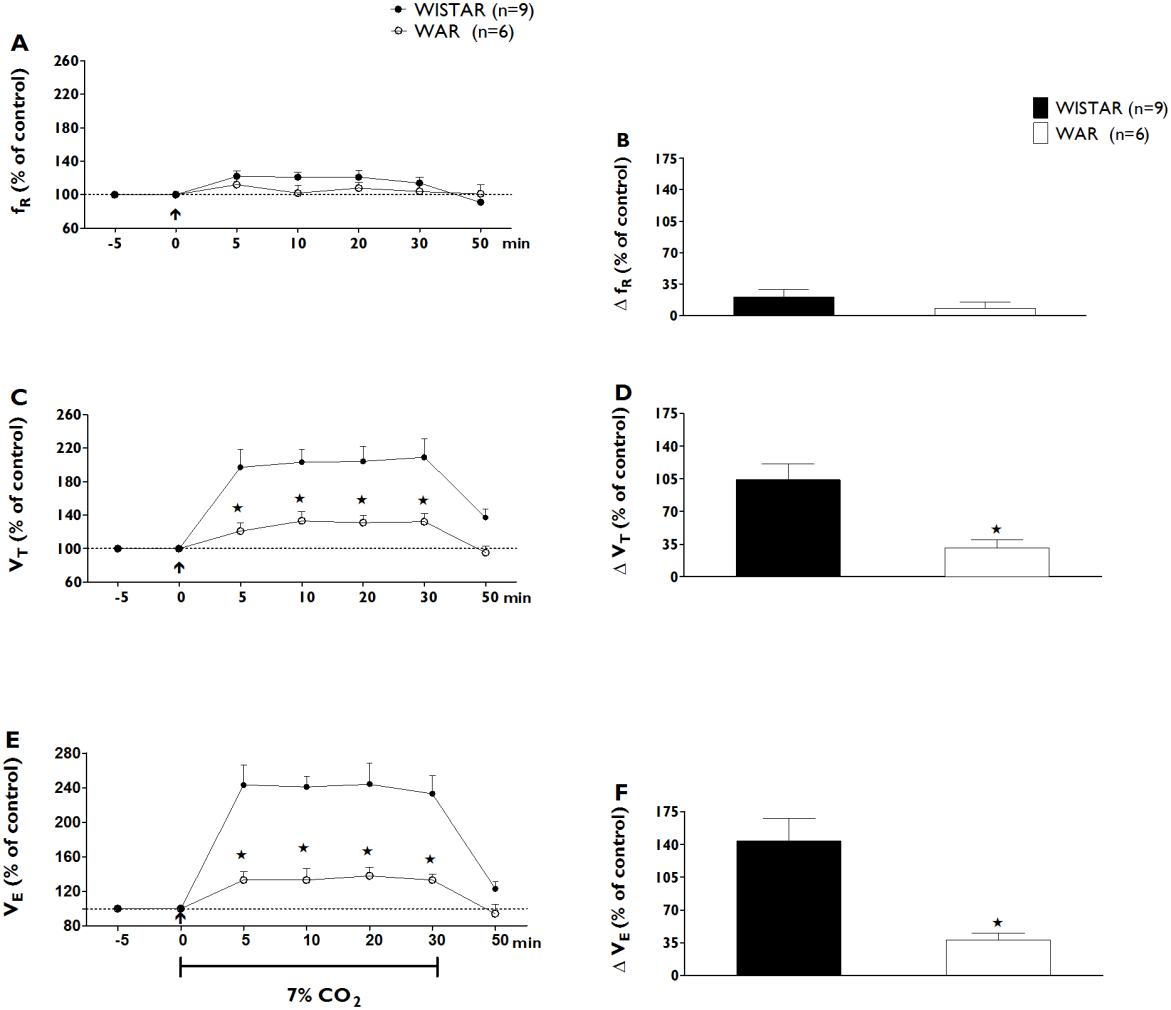
Changes in Respiratory Variables after Hypercapnia. Time-course of respiratory frequency (fR, % of control, A), tidal volume (VT, % of control, C) and minute ventilation (VE, % of control, C) changes during 30 min of hypercapnia (7% CO_2_) in Wistar (blue dots) and WAR groups (red dots). Peak changes in baseline respiratory frequency (ΔfR, % of control, B), tidal volume (ΔVT, % of control, D) and minute ventilation (ΔVE, % of control, F) 20 min after the beginning of hypercapnia (7% CO_2_) in Wistar (blue bars, n = 9) and WAR groups (red bars, n = 6). The arrow indicates the moment of the establishment of hypercapnia. Data shown are expressed as mean ± SEM. p<0.05, Two-way ANOVA followed by Bonferroni’s post-hoc test. (*) Significantly different from Wistar control group.

Hypercapnia caused a significant increase in VE relative to the VE values that were associated with normoxia (the control period) in both groups (P<0.001). This increase resulted mainly from elevations in VT in both the normal Wistar (n = 9) and WAR groups (n = 6, Fig 1). The observed increases in VT and VE in both groups of rats peaked at an average of 20 min (Fig 1, panels D and F, respectively) after the establishment of hypercapnia, and the values returned to baseline levels during the first 20 min after the end of the period of exposure to hypercapnic conditions. However, the average magnitude of the ventilatory response to hypercapnia was significantly greater among animals in the Wistar control group than among animals in the WAR group during minutes 5–30 of the period of exposure to hypercapnia (144 ± 24% vs. 38 ± 7%, p<0.01, Fig 1). Expressing the ventilatory responses to hypercapnia, as either absolute data or relative to the body weight yielded similar results. The Wistar group exhibited greater increases in VE than the WAR group in all analysis (p<0.05, Table 2).

#### Thermoregulatory response to hypercapnia

**Fig 2:**
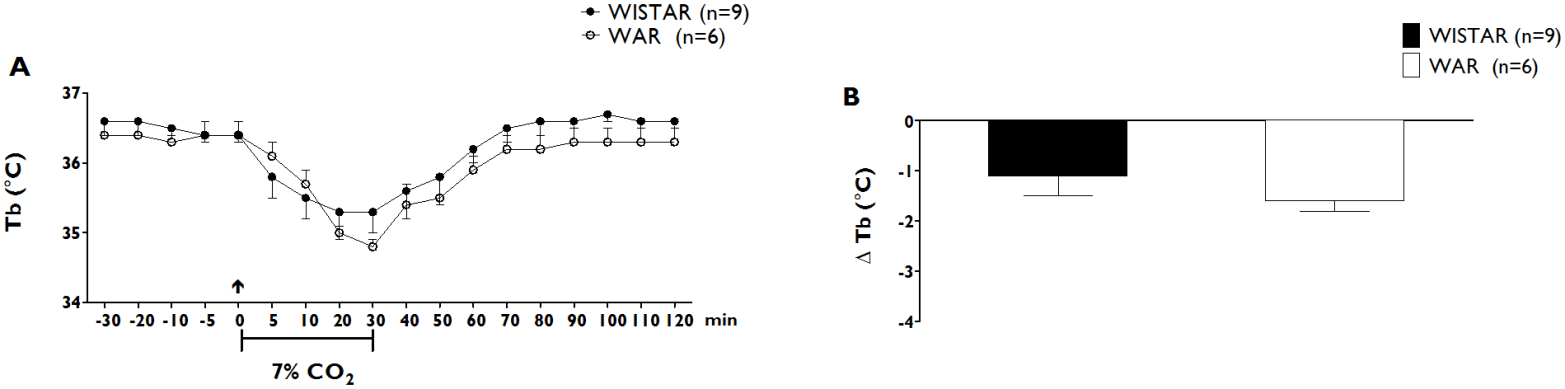
Changes in body temperature after Hypercapnia. Time-course of body temperature (Tb, ΔC, A) changes during 30 min of hypercapnia (7% CO_2_) in Wistar (blue dots) and WAR groups (red dots). Peak changes in baseline body temperature (ΔTb, ΔC, B) 30 after the beginning of hypercapnia (7% CO_2_) in Wistar (blue bar, n = 9) and WAR groups (red bar, n = 6). The arrow indicates the moment of the establishment of hypercapnia. Data shown are expressed as mean ± SEM. p<0.05.

Animals in the Wistar control group (n = 9) displayed a significant drop in Tb during exposure to hypercapnic conditions; this drop in body temperature began 10 min after the establishment of hypercapnia (control: 36.4 ± 0.1°C vs. 10 min after the onset of hypercapnia: 35.5 ± 0.3°C, p< 0.05; Panel A of Fig 2). The average peak of the decrease in the Tb of animals of the Wistar control group occurred 30 min after the onset of exposure to a 7% CO_2_ environment (Panel B of Fig 2) and returned to baseline levels during the first 20 min after the end of the period of exposure to the hypercapnic environment. Animals in the WAR group (n = 6) also had a significant drop in Tb that began 10 min after the establishment of hypercapnic conditions (control: 36.6 ± 0.2 vs. 10 min after the onset of hypercapnia: 35.7 ± 0.2°C, p< 0.05; Panel A of Fig 2). The average peak of the decrease in the Tb of animals in the WAR group also occurred 30 min after the initiation of exposure to a 7% CO_2_ environment (Panel B of Fig 2). There were no significant differences in magnitude of the Tb responses to hypercania between Wistar and WAR groups (Wistar: -1.1 ± 0.4 vs WAR: -1.6 ± 0.2, p>0.05; Panel B of Fig 2).

#### Ventilatory responses to hypoxia

**Fig 3:**
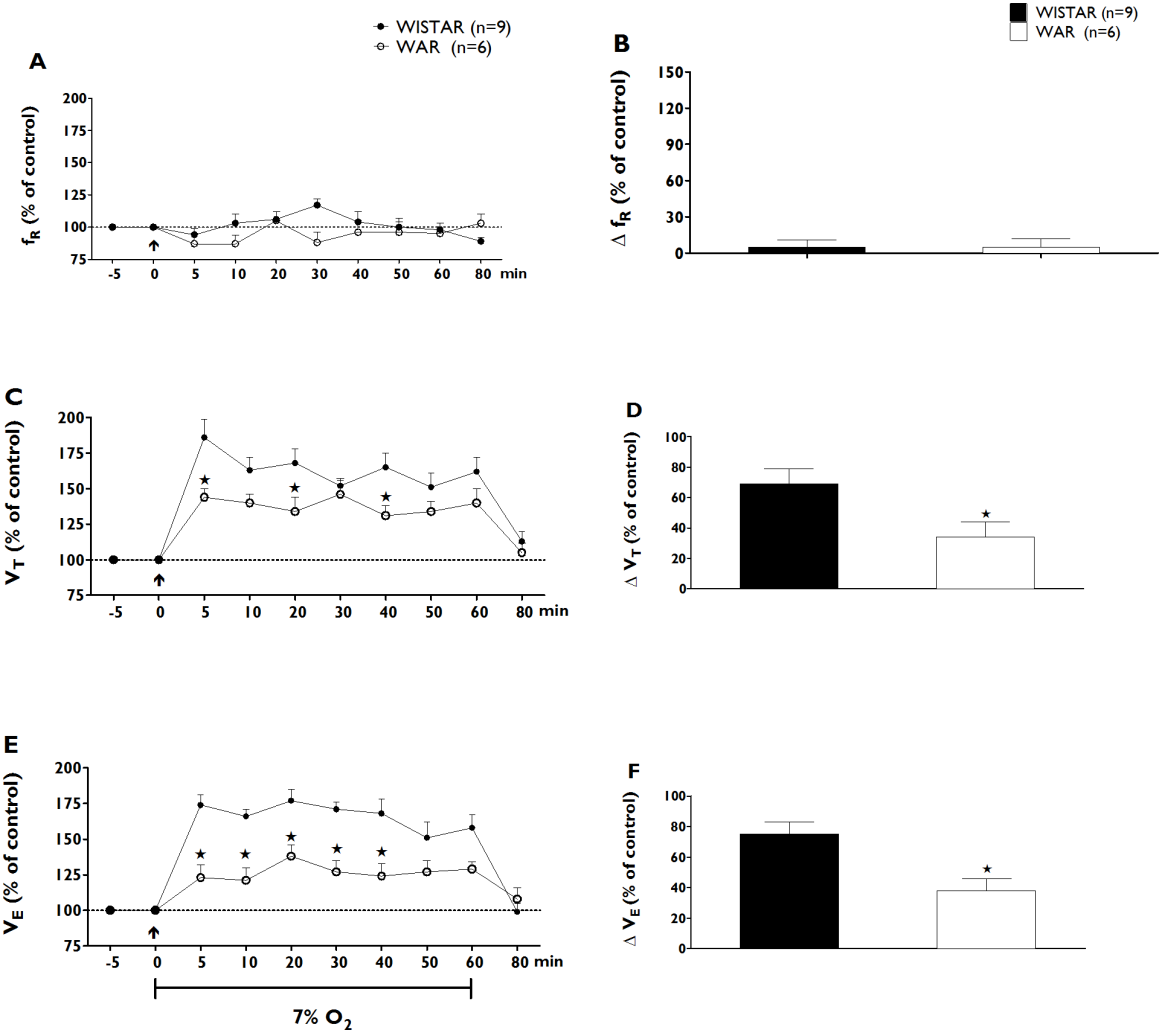
Changes in Respiratory variables after Hypoxia. Time-course of respiratory frequency (fR, % of control, A), tidal volume (VT, % of control, panel C) and minute ventilation (VE, % of control, E) changes during 60 min of hypoxia (7% O_2_) in Wistar (blue dots) and WAR groups (red dots). Peak changes in baseline respiratory frequency (ΔfR, % of control, B), tidal volume (ΔVT, % of control, panel D) and minute ventilation (ΔVE, % of control, F) 20 min after the beginning of hypoxia (7% O_2_) in Wistar (blue bars, n = 9) and WAR groups (red bars, n = 6). The arrow indicates the moment of the establishment of hypoxia. Data are expressed as mean ± SEM. p<0.05, Two-way ANOVA followed by Bonferroni’s post hoc test. (*) Significantly different from Wistar control group.

Exposure to hypoxia also caused a significant increase in the average VE values of both groups (p<0.001), and the observed increases in VE resulted from increases in the VT values of both groups (Fig 3). In both groups, the increases in the values of VE peaked at an average of 20 min after the onset of hypoxia, and the VT and VE values returned to baseline levels within 20 min of the end of the period of exposure to hypoxia. As in the hypercapnic conditions, the ventilatory response to hypoxia in the control group of Wistar rats (n = 9) was significantly higher than in animals in the WAR group (n = 6) after 20 min of exposure to hypoxic conditions (177 ± 8% vs. 138 ± 8%; panel F of Fig 3). Similarly to hypercapnia, expressing the ventilatory responses to hypoxia as to the body weight and absolute values yielded similar results. The Wistar group exhibited greater increases in VE than the WAR group in all analysis (p<0.05, Table 2). Together, these results shown that exposure to both hypercapnic and hypoxic conditions resulted in attenuated ventilatory responses in WAR animals.

#### Thermoregulatory responses to hypoxia

**Fig 4:**
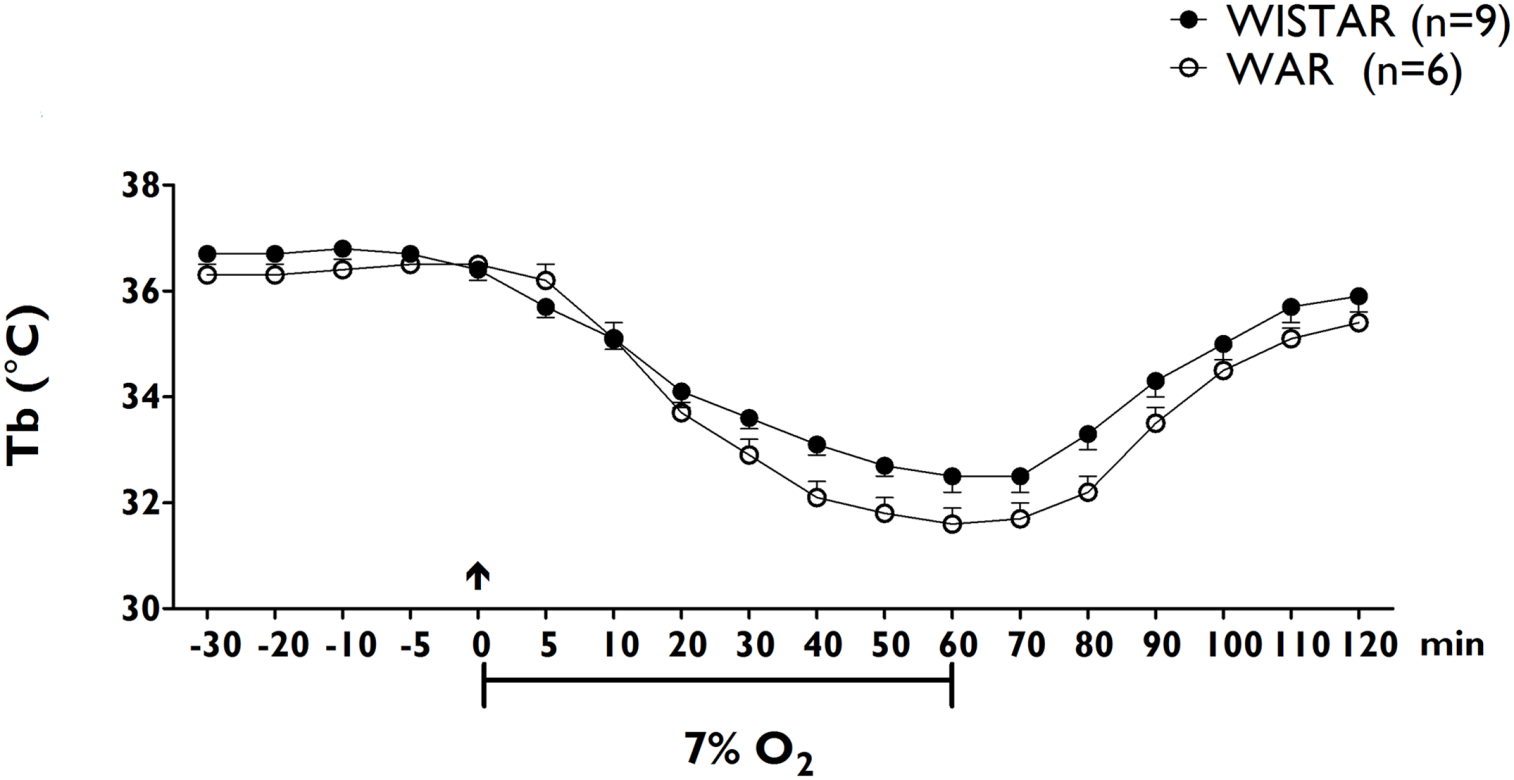
Changes in body temperature after Hypoxia. Time-course of temperature body (Tb, ΔC, panel A) changes during 60 min of hypoxia (7% O_2_) in Wistar (blue dots) and WAR groups (red dots). Peak changes in baseline body temperature (ΔTb, ΔC, panel B) 60 min after the establishment of hypoxia (7% O_2_) in Wistar (blue bar, n = 9) and WAR groups (red bar, n = 6). The arrow indicates the moment of the establishment of hypoxia. Data are expressed as mean ± SEM. p<0.05.

Animals in the Wistar control group displayed a significant drop in Tb that began 10 min after the establishment of hypoxia (control: 36.4 ± 0.2°C vs. 10 min after the onset of hypoxia: 35.1 ± 0.3°C, p< 0.05; Panel A of Fig 4). The average peak of the decrease in the Tb of the Wistar group animals occurred 60 min after the onset of exposure to hypoxia (Panel B of Fig 2), and the Tb of these animals returned to baseline levels within 60 min after the end hypoxia exposure. Animals in the WAR group also displayed a significant drop in Tb that began 10 min after the establishment of hypoxia (control: 36.5 ± 0.1°C vs. 10 min after the onset of hypoxia: 35.1 ± 0.3°C, p< 0.05; Panel A of Fig 4). The average peak of the decrease in the Tb of the animals in the WAR group also occurred at 60 min after the onset of exposure to a 7% O2 (Panel B of Fig 2). Similarly to hypercapnia, there were no significant differences in magnitude of the Tb responses to hypoxia between Wistar and WAR groups (Wistar: -4.2 ± 0.3 vs WAR: -4.7 ± 0.2, p>0.05; Panel B of Fig 4).

All raw data on thermoregulatory and ventilatory responses to both acute hypercapnia and acute hypoxia, when WAR and Wistar animals were compared, are in Supporting Information (S1 and S2 Datasets).

### Behavioral Analysis

**Fig 5:**
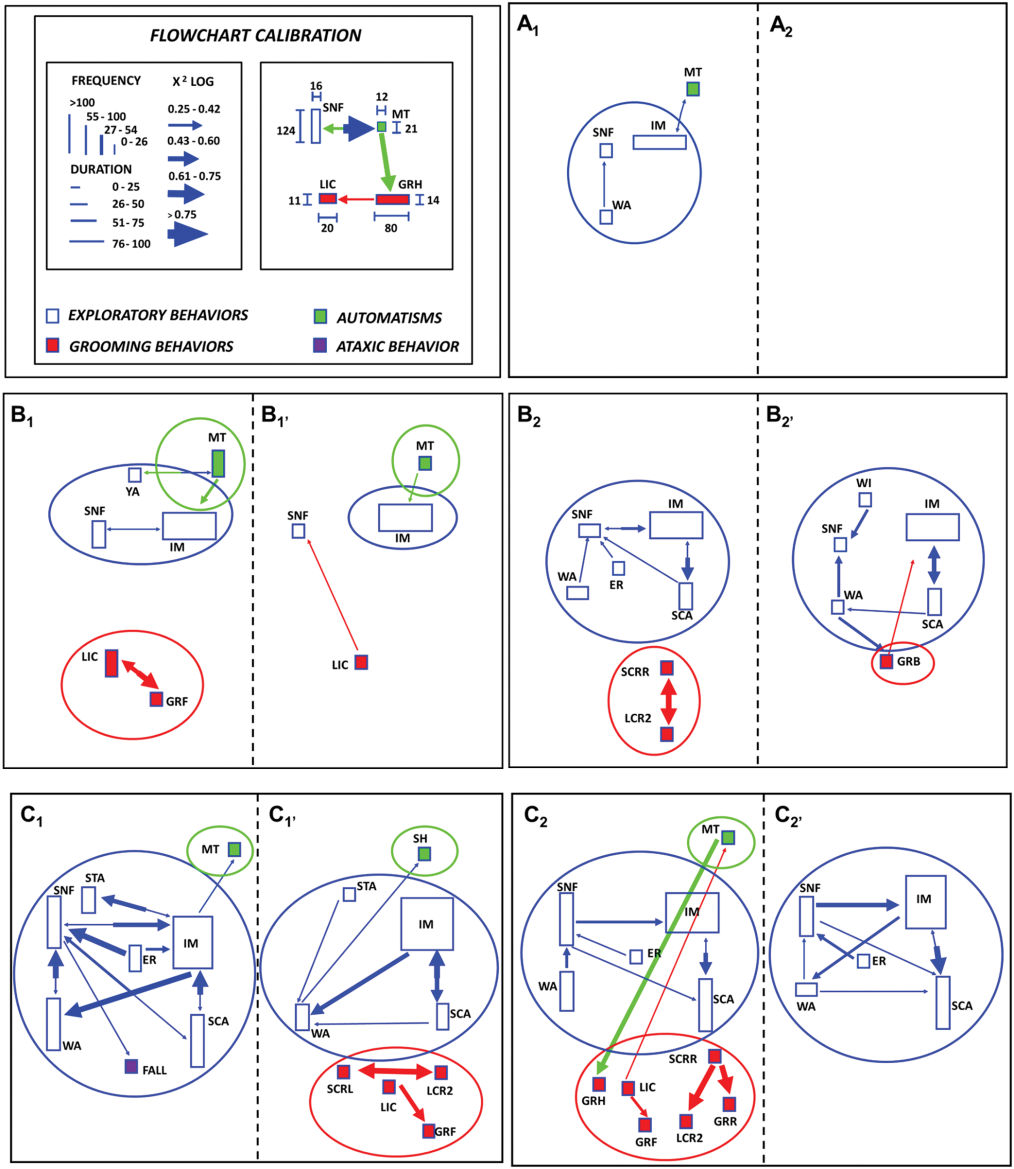
Neuroethological analysis and a detailed glossary help in the detection of features of behavior under hypercapnia and hypoxia conditions. Standard flowchart and calibration parameters (top left) used to represent behavioral sequences using the neuroethological approach [10, 15]. Heights of rectangles represent behavior frequency, while bases of rectangles represent behavior duration in the observation periods. Codes in rectangles represent behavioral items. Circles/ellipses identify qualitative grouping of main behavioral clusters such as facial automatisms (blue open symbols), exploration (green filled symbols) and grooming behavior (red filled symbols). Arrows represent statistical values (Chi-square) measuring degrees of association between pairs of behaviors (dyads) in a given sequence. Behavioral sequences observed during normoxia (control period) in Wistar (A_1_) and WAR groups (A_2_). Behavioral sequences observed during hypercapnia in Wistar (B_1_ and B_1_’) and WAR groups (B_2_ and B_2_’). Behavioral sequences observed during hypoxia in Wistar (C_1_ and C_1_’) and WAR groups (C_2_ and C_2_’). In all cases Wistar (n = 9) and WAR (n = 6). Dictionary of behaviors: ER, erect posture; FALL, falling; GRB, grooming of body; GRF, grooming of face; GRR, grooming of body (right); IM, immobility; LIC, licking of claws; LCR2, licking of claws (right posterior); MT, masticatory movements; SCA, scanning; SH, head shaking; SCRR, scratching of body (right); SNF. sniffing;WA; walking; WI, Withdrawing; YA, yawning. **Observation:** The presence of falling (FALL) is frequent in limbic seizures or as an ataxic posture. However, it was present here in one Wistar animal submitted to hypoxia (C_1_) and we have no clear explanation for that.

Five observation windows from different experimental periods are represented in Fig 5 for both Wistar rats (A_1_: normoxia; B_1_: min 0–5 of exposure to hypercapnia; B_1_’: min 25–30 of exposure to hypercapnia; C_1_: min 0–5 of exposure to hypoxia; C_1_’: min 55–60 of exposure to hypoxia) and WARs (A_2_: normoxia; B_2_: min 0–5 of exposure to hypercapnia; B_2_’: min 25–30 of exposure to hypercapnia; C_2_: min 0–5 of exposure to hypoxia; C_2_’: min 55–60 of exposure to hypoxia).

#### Normoxia (Fig 5A)

During the control period rats in the Wistar group (A_1_) showed a modest cluster of exploratory behaviors including SNF, WA and IM (blue symbols, see dictionary of behaviors in Fig 5 legend), and some animals also showed evidence of weak interactions with orofacial automatisms (MT, green symbol). However, no behavioral interactions were observed among animals in the WAR group during this control period (A_2_).

#### Hypercapnia (Fig 5B)

During the initial hypercapnia period (0–5 min, B_1_), there is a similar basic pattern of exploration behaviors among animals in the Wistar control group (blue symbols), in comparison to the behavior patterns of the Wistar animals during baseline window (normoxia period, A_1_). Moreover, the exploratory behaviors maintain a partial interaction with orofacial automatisms (MT, green symbol, B_1_). Interestingly, the Wistar rats also began to demonstrate a new cluster of grooming behaviors (red symbols, B_1_) during this initial period of hypercapnia. However, when the behaviors of the Wistar control rats during the final minutes of the hypercapnia period (25–30 min, B_1_’) are evaluated, it is possible to observe that these animals showed a reduction in the frequency and duration of their interactions with the orofacial automatisms (MT, green symbol, B_1_’), and there was a significant reduction of grooming and exploratory behavior clusters. Notably, MT still interacts with IM (long duration) (blue symbol, B_1_’).

With respect to the hypercapnia-induced behavioral responses among animals in the WAR group (Fig 5B) during the first 5 min of exposure to a 7% CO_2_ environment (min 0–5, B_2_), these rats showed a clear cluster of exploratory behaviors (blue symbols), particularly when compared with the exploratory behaviors of animals in the Wistar control group (B_1_). Moreover, the presence of strong interactions among all exploratory behaviors (SNF, IM, WA, ER and SCA) can easily be observed in WARs. Similar to animals in the Wistar rat control group (B_1_), WAR also showed a cluster of grooming behaviors during the initial period of exposure to hypercapnia (red symbols, B_2_). During the last 5 min of exposure to hypercapnia, however, and in contrast to animals in the Wistar group (B_1_’), both the strong cluster of exploratory behaviors (SNF, IM, WA, SCA and WI, blue symbols) and the interactions among them persisted among animals in the WAR group (B_2_’). In addition, during this final period, WAR group animals (B_2_’) displayed GRB as a single grooming behavioral item (GRB; l) which interacted with the IM (exploratory behavior; blue symbol).

#### Hypoxia (Fig 5C)

At time 0–5 min of exposure to a 7% O_2_ environment, rats in the Wistar control group (C_1_) showed an important cluster of exploratory behaviors that was characterized by both the presence of SNF, STA, WA, ER, IM, and SCA behaviors (blue symbols) and the presence of strong interactions among them. In addition, the IM exploratory behavior (blue symbol, C_1_) interacted with orofacial automatisms (MT, green symbol, C_1_). Linked to SNF from the exploratory cluster it appeared falling (FALL; purple symbol), a behavioral item rarely seen in the context of exploration, and usually associated to seizures and ataxia. It was also observed that the frequencies, interactions and durations of exploratory behaviors were significantly attenuated during the final minutes of the hypoxic stimulation of the Wistar group (55–60 min, C_1_’), and the Wistar rats only displayed a cluster of grooming behaviors including SCRL, LCR2, LIC and GRF (red symbols, C_1_’) during this final period of exposure to hypoxia. We observed that during the first 5 min of exposure to hypoxia (time 0–5 min of the hypoxic period) (C_2_), animals in the WAR group showed a significant cluster of exploratory (SNF, WA, ER, IM and SCA, blue symbols) and grooming behaviors (GRH, LIC, SCRR, GRF, LCR2 and GRR, red symbols) with clear interactions among the various behaviors. The orofacial automatism (MT, green symbol, C_2_) interacted bidirectionally with some of the aforementioned grooming behaviors (red symbols, C_2_). It is important to note that the cluster of grooming behaviors was absent among animals in the control Wistar group during this initial hypoxic window (time 0–5 min) (C_1_). At the end of the period of exposure to hypoxia (min 55–60), animals in the WAR group (C_2_’) continued to display a cluster of exploratory behaviors (SNF, WA, ER, IM and SCA), whereas evidence of grooming behaviors among these animals had completely disappeared. Two additional events in this period were the appearance in WARs of the automatism shaking head (SH) behavior interacting with the exploratory behavior scanning (SCA) and the increase of the frequency of immobility (IM).

## Discussion

This is the first study addressing an integrated response of ventilation, body temperature and behavioral alterations in a genetic model of epilepsy (the WAR strain) exposed to 7% hypoxia and 7% hypercapnia. No significant difference was observed between the WAR and Wistar control group with respect to the thermoregulatory response elicited by exposure to both acute hypercapnia and acute hypoxia. However, exposure to both hypercapnic and hypoxic conditions resulted in attenuated ventilatory responses in WAR animals. With respect to behavioral responses, the present study showed that our genetic model of epilepsy has an altered ability to react to (or compensate for) changes in PCO_2_ or PO_2_.

Our data offered evidence of differences in the hypercapnia- and hypoxia-induced responses of control Wistar rats and WARs. In fact, during exposure to each of these environments, the Wistar rats exhibited greater increases in VT and VE than the WARs; the ventilatory responses to 7% CO_2_ or 7% O_2_ exposure appeared to be compromised in WARs. These observations indicated that our genetic model of epilepsy [9] has an altered ability to compensate for changes in arterial blood gas levels with changes in respiration that may be specific to both an increase in PaCO_2_ or a decrease in PaO_2_. These changes probably involve an imbalance of central and peripheral chemoreceptors and may be a limiting factor in the generation of adequate respiratory adjustments in response to hypercapnic or hypoxic events.

There are several converging lines of evidence that genetic defects in the serotonin (5-HT) system are linked to the respiratory alterations that are observed during seizures [18,19]. For example, transgenic mice that carry a deletion of the 5-HT 2_C_ receptor and that are susceptible to AS experience post-ictal respiratory arrest that invariably leads to death if the animals are not resuscitated [20]. Another link between respiratory alterations and the 5-HT receptor have been also established in DBA/2 mice, (also susceptible to AS); increasing the level of 5-HT prevents seizure-induced respiratory arrest in these animals, whereas blocking 5-HT receptors increases the likelihood of it [19,21]. Moreover, under non-pathological condition, evidence from several studies indicates that medullary 5-HT neurons posse properties that qualify them as being appropriate candidates for central respiratory chemoreceptors [16,22,23,24]; these neurons also play an important role in the interaction between central and peripheral ventilatory responses to hypercapnia [25]. On the basis of data from the existing literature, it is therefore reasonable to hypothesize that the reduced ventilatory responses that we observed in the WARs in our study may be linked to defects in the 5-HT system. Like the changes in ventilatory responses, exposure to hypercapnia or hypoxia also induces significant decreases in the Tb of many species [26].

To the best of our knowledge, no study has systematically assessed the hypercapnia- or hypoxia-induced behavioral responses in Wistar or WAR animals. We accomplished this goal in the present study by using a neuroethological method that was based on flowchart depictions of behavioral sequences, a method that has been extensively used in our laboratory to analyze behavioral sequences in experimental epilepsy [10] and in epilepsy patients [27,28]. Using this approach, we observed that exposure to hypercapnia or hypoxia induced differential temporal expression patterns of three relatively conspicuous behavioral clusters in both normal Wistar rats and WARs. The behavioral clusters included exploratory behaviors (blue open symbols), orofacial automatisms (green filled symbols), and grooming behaviors (red filled symbols), and the frequencies, latencies and durations of these behavioral responses were significantly different between the two groups of animals, suggesting important differences in the degrees to which WAR and Wistar animals are able to respond to changes in PCO_2_ or PO_2_.

During the initial phase of exposure to a 7% CO_2_ (0–5 min) environment, animals in both the Wistar and WAR groups displayed two important behavioral clusters: exploratory behaviors and grooming behaviors. Moreover, animals in the Wistar group also showed MT orofacial automatisms during this phase. At the end of the period of exposure to a hypercapnic environment (min 25–30), however, the Wistar rats appear to have adapted to the 7% CO_2_ atmosphere; both the grooming and exploratory behavior clusters disappeared (only the IM behavior remained), and the frequency of MT orofacial automatisms had decreased significantly. In contrast, animals in the WAR group still demonstrated strong clusters of exploratory and grooming behaviors during the same temporal window.

As in hypercapnic conditions, animals in both the normal Wistar and WAR groups showed clear clusters of exploratory behaviors and orofacial automatisms during the first 5 min after the onset of exposure to the hypoxic environment. However, although the WAR maintained the exploratory behavior cluster, the behavioral responses of the Wistar rats were significantly decreased during the last few minutes (min 55–60) of exposure to a hypoxic environment. This suggests that normal Wistar animals were also able to adapt to a hypoxic situation and this phenomenon may be associated with the central and peripheral chemoreceptors of these animals being able to efficiently induce significant increases in ventilation during hypercapnia or hypoxia. It is obvious that the two strains of rats are using different strategies to cope with the environmental changes in PCO_2_ or PO_2_.

Another important behavioral difference between the animals in the Wistar and WAR groups was the hypoxia-induced latency before the initiation of grooming behavior. In contrast to animals in the Wistar group, 5 min of exposure to a 7% O_2_ environment were sufficient to induce a more prominent grooming behavior among the WAR animals. Several studies have indicated that grooming behaviors are most commonly induced in anxiogenic situations and situations in which conflict is present [29]. In addition, another study suggested that some grooming patterns can be used as behavioral markers of stress in rats [30]. Thus, the acute hypergrooming response displayed by WARs in the present study suggests that these animals experienced a higher level of stress than animals in the Wistar group. These data are in accordance with the results of previous studies from our laboratory; in those studies, we demonstrated that in addition to being genetically susceptible to AS, WARs are generally more anxious than AS-resistant Wistar rats [31]. Thus, we cannot discard the possibility that the observed increase in grooming behavior may have anxiolytic properties.

The WAR strain represents a quite important model of epilepsy [9] and associated comorbidities such as increased stress [31], hypertension, tachycardia and increased sympathetic tonus [32] and hyperactivity of the HPA axis associated to adrenal gland hyperplasia [17]. As an important addition to this characterization, the current study represents an effort to better describe the behavioral, respiratory and thermoregulatory responses of WARs to both hypercapnia and hypoxia. The present data will be of great value in the understanding of breathing dysfunctions that may occur during seizure events, probably coupled to neuroanatomical substrates for the attenuated respiratory responses. In this context, interesting questions remain open: As hypothesized:

1. Are the 5-HT system dysfunctions underlying the depressed respiratory responses observed presently?
2. Where in the brainstem does the dysfunction occur?
3. Is it only restricted to medullary raphe region, which has a considerable amount of 5-HT neurons involved in respiratory control network?
4. As CO_2_ central chemoreception is being considered a widely distributed function in the brain [33,34]; does WAR have alterations in other CO_2_-sensitive regions such as the retrotrapezoid nucleus, locus coeruleous, nucleus tractus solitarii?
5. Does WAR have altered peripheral-central chemoreceptors interaction?

Answering these questions will provide important information to be added to previous data on respiration-epilepsy coupling.

In fact, we preliminary investigated the respiratory central chemoreflex as well as the role of serotonergic neurotransmission within the brainstem in WARs, and we found that these animals have reduced resting minute ventilation [35]. Additionally, we also noted that WARs have reduced minute ventilation elicited by central chemoreflex activation (hypercapnia—7% CO2) and that the 5-HT innervation to the brainstem (originated in the dorsal raphe nucleus) was reduced in WARs. Furthermore, we also showed that the ventilatory response to 5-HT within the brainstem was significantly reduced in awake WARs. Our results suggest a respiratory disorder, as well as a reduction of the 5-HT neurotransmission within the brainstem [35], which, together with those on endogenous adrenal, sympathetic and behavioral alterations discussed in the current study, all compatible with WARs as a more stressed strain, strongly give support to the use of this genetically-developed model as suitable to further study the mechanisms associated with SUDEP. In fact, some of the SUDEP models have shown not only respiratory failure but also 5-HT alterations [36] and recent experiments have shown how brainstem chemoreception and 5HT neurotransmission are critical components of a protection mechanism against SUDEP [37].

In conclusion, WAR animals have attenuated ventilatory responses during exposure to both hypercapnia and hypoxia, and these altered responses were accompanied by altered behavioral responses. These results potentially facilitate future studies with WARs, aimed at elucidating and model the impact of the high risk breathing disturbances that are sometimes observed among patients with epilepsy.

## Supporting Information

S1 Dataset First block of raw data on thermoregulatory and ventilatory responses to both acute hypercapnia and acute hypoxia, when WAR and Wistar animals were compared. (XLS)

S2 Dataset Second block of raw data on thermoregulatory and ventilatory responses to both acute hypercapnia and acute hypoxia, when WAR and Wistar animals were compared. (XLS)
